# Study on the Current Status of Supportive Care Needs of Elderly Breast Cancer Patients and Influencing Factors

**DOI:** 10.1155/nrp/2880186

**Published:** 2026-07-22

**Authors:** Weishuang Tian, Junxiong Gao, Tingting Wei, Yan Zhang, Xinyu Liu, Xia Li

**Affiliations:** ^1^ The Third Department of Breast Surgery, Tianjin Medical University Cancer Institute and Hospital, National Clinical Research Center for Cancer, Tianjin Key Laboratory of Cancer Prevention and Therapy, Tianjin’s Clinical Research Center for Cancer, Tianjin, China, tmucih.com; ^2^ Digital & Information Technology Center, Tianjin Pharmaceutical Da Ren Tang Group Corporation Limited, Tianjin, China

## Abstract

**Aim:**

This study investigated the current status and key influencing factors of supportive care needs among elderly breast cancer patients, with a focus on identifying critical determinants through a survey and advanced machine learning techniques.

**Design:**

The cross‐sectional study randomly interviewed 180 elderly breast cancer patients, and 157 valid observations were retained for analysis.

**Methods:**

Firstly, patients were surveyed by two questionnaires. One is for fundamental information, which includes demographics, clinical, and psychosocial variables; the other is the Chinese version of the Supportive Care Needs Survey‐Short Form 34 (SCNS‐SF34). And then, the questionnaires’ results were transferred into a tabular dataset, with each question from the questionnaires serving as a column in the dataset. Secondly, an Extreme Gradient Boosting (XGBoost, a supervised learning algorithm) model was trained on the dataset. Fundamental features served as input variables, and the label was the total score of SCNS‐SF34. Thirdly, the Shapley Additive Explanations (SHAP) framework was applied to interpret the XGBoost model by quantifying feature importance.

**Results:**

The XGBoost model demonstrated moderate predictive efficacy (*R* ^2^ = 0.58). After adopting the SHAP method on the model, we have the following findings. Fatigue severity emerged as the most significant feature of increased care needs, followed by weight, age, and educational level. Surgical interventions and low exercise frequency were associated with heightened demands, while managed chronic conditions (e.g., hypertension) and higher socioeconomic status (e.g., pension‐based income) mitigated the care needs. Psychosocial factors, such as minority ethnicity and low health literacy, exacerbated unmet needs, whereas regular family support and higher education levels buffered demands.

**Conclusion:**

This study highlights the multidimensional nature of supportive care needs in elderly breast cancer patients, emphasizing the interplay of physiological, treatment‐related, and psychosocial factors. The findings suggest that targeted interventions, such as fatigue management, culturally sensitive care navigation, and structured support for low‐literacy populations, are essential to address unmet needs. Future research should prioritize longitudinal designs and policy integration to enhance care delivery and promote health equity.

**Reporting Method:**

The study adhered to STROBE guidelines for cross‐sectional studies to ensure transparent and comprehensive reporting of results.

**Patient or Public Contribution:**

Not applicable.

## 1. Introduction

Breast cancer remains the most common malignancy in women globally, with 2.3 million new cases reported in 2020, accounting for 11.6% of all cancers and ranking first among female malignancies [1]. It is increasingly emerging as a geriatric disease, with a median age at diagnosis of 62 years. Notably, 84% of invasive breast cancer cases and 91% of breast cancer‐related deaths occur in women aged ≥ 50 years, including 52% of fatalities in women aged ≥ 70 years [2]. Understanding the supportive care needs of elderly breast cancer patients is critical, as these needs span physical symptoms (e.g., fatigue), psychological distress (e.g., anxiety), and social barriers (e.g., lack of family support). Despite its recognized importance, detailed exploration into the specific needs and influencing factors for elderly breast cancer patients remains limited.

Present studies emphasized that elderly breast cancer patients have diverse and complex supportive care needs, with variations influenced by healthcare service satisfaction, tumor location, caregiver roles, and socioeconomic factors [3–5]. Additionally, individual psychological characteristics are pivotal in driving higher‐intensity, longer‐duration supportive care needs among the patients [6].

Meanwhile, based on three recent systematic reviews, we identified key limitations in existing research on breast cancer patients’ supportive care needs: First, Reid‐Agboola confirmed the utility of Comprehensive Geriatric Assessment (CGA) in aiding treatment decisions for older breast cancer patients, yet most studies included in this review were conducted in Europe, with a notable lack of relevant data from Asian populations [7]. Second, Nguyen reported that 84% of postdischarge breast cancer patients had at least one unmet supportive care need [8]; however, the majority of these studies adopted cross‐sectional designs and conventional regression analyses, limiting the exploration of complex associations. Third, Khajoei highlighted the pervasiveness of supportive care needs among breast cancer survivors [9], but the review revealed a lack of targeted subgroup analysis specific to the elderly population among the included studies.

To directly address these limitations, this study first recruited elderly Chinese breast cancer patients to provide much‐needed evidence on their supportive care needs, filling a critical gap in the literature on Asian populations. Second, instead of conventional regression analyses, we adopted an interpretable machine learning framework integrating Extreme Gradient Boosting (XGBoost) and SHapley Additive Explanations (SHAP) to capture complex nonlinear relationships and interaction effects among multiple predictors. Third, by exclusively focusing on patients aged 60 years or older, we conducted a nuanced investigation into age‐specific supportive care needs and their underlying determinants. Through comprehensive analysis, we aimed to identify key determinants of these needs and inform targeted, personalized interventions, thereby improving the quality of life and health outcomes for this vulnerable group. This research not only fills the identified literature gaps but also provides actionable clinical insights to optimize geriatric breast cancer care services.

## 2. Background

Breast cancer, one of the most common malignancies in women, poses unique challenges for elderly patients, profoundly affecting their physical, psychological, and social well‐being [10]. In China, the incidence and mortality of female breast cancer have shown distinct epidemiological trends, with studies reporting age‐standardized incidence rates of 27.8 per 100,000 women [11]. Recent reviews further highlight that risk factors such as delayed diagnosis and limited access to specialized care exacerbate the burden of breast cancer in elderly populations [4]. These studies underscore the urgency of addressing supportive care needs in this vulnerable group.

Supportive care needs among elderly breast cancer patients are multifaceted, encompassing physical symptom management, psychological support, and socioeconomic assistance, with cultural and systemic barriers exacerbating disparities in clinical trial participation [12, 13]. For instance, qualitative studies reveal that postmastectomy psychological distress significantly impacts patients’ quality of life [14], while structured interventions, such as home‐based care plans, have demonstrated efficacy in reducing unmet needs during chemotherapy [15]. Furthermore, validated assessment tools like the Supportive Care Needs Survey‐Short Form 34 (SCNS‐SF34) have been critical in quantifying these needs across diverse patient subgroups [16, 17]. We adopted the Chinese version of SCNS‐SF34 in this research. Cancer‐related fatigue was prioritized in this study due to its high prevalence, long persistence [18], and frequent underdiagnosis and inadequate management [19], in contrast to more standardized management of symptoms like pain and nausea. Anxiety, depression, and nausea were not focused on, mainly due to research focus and resource constraints, as their incidence in breast cancer patients is lower than that of fatigue [20].

Furthermore, the influences of single factors, such as clinical or demographic variables, on the supportive care needs have been studied; in contrast, the interplay between physiological, treatment‐related, and psychosocial dimensions remains under‐investigated. By integrating analytical techniques, such as machine learning, with robust interpretability tools like SHAP [21], this study aims to provide a more holistic understanding of the factors influencing care needs in elderly breast cancer patients.

SHAP has notable methodological advantages: rooted in the axiomatic system of Shapley values, it ensures fair and unbiased feature contribution allocation in multivariable interaction scenarios [22]; it enables both global feature importance ranking and individual sample‐level feature impact quantification; and it exhibits strong robustness in handling feature collinearity with model‐agnostic universality. SHAP has been widely validated in clinical oncology research, including the prediction of postoperative length of stay after gastrectomy [23] and interpretable breast cancer classification with XGBoost [24, 25], which confirms its clinical applicability and reliability for analyzing cancer patient‐related predictive models.

This research is particularly timely given the increasing emphasis on personalized medicine and patient‐centered care. By identifying key determinants of supportive care needs, this study seeks to inform targeted interventions that address the unique challenges faced by elderly breast cancer patients, ultimately enhancing their quality of life and reducing the burden on healthcare systems.

## 3. The Study

This study strictly followed the ethical principles outlined in the Declaration of Helsinki. The research protocol was reviewed and approved by the Ethics Committee of Tianjin Medical University Cancer Institute and Hospital (ethics approval number: bc20252552). All participants were clearly informed of the research objectives, survey content, data usage, voluntary participation principle, and the right to refuse participation or withdraw from the study at any time without any negative consequences. Prior to data collection, written informed consent was obtained from all elderly breast cancer patients enrolled in this study. All personal and clinical information was anonymized, and all raw data were kept strictly confidential and used only for academic research purposes in order to protect patients’ privacy and information security.

### 3.1. Aim

Our research team is from a leading specialized cancer hospital in China. The team’s overarching objective is to elevate the quality of life and optimize care outcomes for elderly breast cancer patients. To reach this ultimate goal, we conduct a series of studies, which consisted of three phases.

1. Investigate the current status and key influencing factors of supportive care needs among elderly breast cancer patients.
2. Utilize existing assistant tools such as interactive toys integrated with LLMs for communication to help address the supportive care needs of elderly breast cancer patients.
3. Develop a Family‐Community‐Hospital collaborative model, to long‐term improve the supportive care level for patients.

This present study is part of the first phase.

### 3.2. Design

**FIGURE 1:**
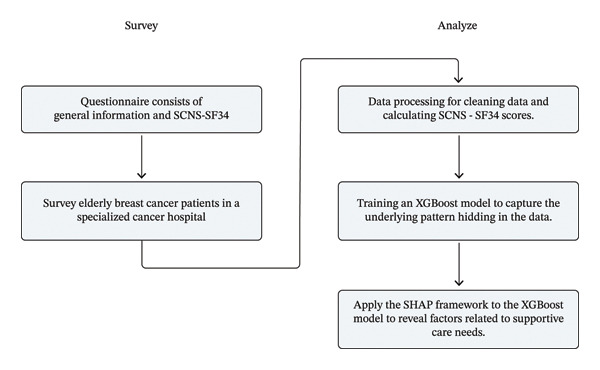
SCNS‐SF34 survey and XGBoost–SHAP analysis process.

This study comprised two phases: systematic data collection through a survey, followed by analysis using statistical and machine learning methods (see Figure 1).

To identify key factors influencing supportive care needs, the XGBoost model and SHAP framework were used in this research. The XGBoost‐SHAP combination is widely applied in medical studies for capturing the underlying patterns and interpreting feature importance [26–28].

XGBoost was employed due to its robustness in modeling nonlinear relationships and processing high‐dimensional data. The algorithm’s capabilities have been validated across diverse domains [29–31]. We trained an XGBoost model to investigate the associations between the patients’ fundamental features and their SCNS‐SF34 total score.

Furthermore, the SHAP framework was applied to quantify feature contributions within the XGBoost model. For each model’s output, this framework assigns a SHAP value to every feature based on its specific value in that instance. Specifically, this value represents the marginal impact of the corresponding feature value on the model’s output. SHAP values then allow us to analyze the influence of patients’ fundamental features on their SCNS‐SF34 total score. Notably, several related studies have employed the SHAP method in breast cancer‐related research References [32–34].

### 3.3. Survey

We administered 180 questionnaires to elderly breast cancer patients. The questionnaire comprised two sections: one for the patients’ fundamental features and another for supportive care needs evaluation. The first section, custom‐designed for this study, collected the patients′ fundamental features (e.g., age groups, income levels, and education levels). To assess supportive care needs, the second section utilized the SCNS‐SF34, selected for its validated multidimensional framework, cultural relevance, and established psychometric robustness among oncology populations [35].

#### 3.3.1. Fundamental Features

The fundamental features questionnaire addressed two main domains, base information and clinical information.

Base information consists of three parts. First, demographic characteristics included personal attributes (e.g., age, weight, and ethnicity), marital status, and living arrangements (e.g., ‘with spouse’ and ‘alone’). Second, socioeconomic factors encompassed residence type, educational attainment (e.g., ‘primary school or below’ and ‘junior college or above’), medical payment methods (e.g., self‐pay and public‐funded), details of monthly income (such as 1000–3000 yuan) and its sources (e.g., pension and children’s support), and family support, as indicated by children’s visitation frequency (e.g., daily and rarely). Third, health‐related indicators quantified exercise frequency (with options like ‘never’, ‘1–2 times/week’, and ‘≥ 5 times/week’) and fatigue severity, measured on a 10‐point scale (0 = no fatigue to 9 = most severe). All data for these fundamental features were gathered using closed‐ended questions with mutually exclusive responses.

The clinical section systematically documented disease progression and management via structured nurse‐reported items. This encompassed disease characteristics, including definitive tumor staging (TNM classification, Stages I–IV), precise anatomic localization (e.g., left, right, or bilateral involvement), and assessment of metastatic spread (yes/no). It also covered treatment details and outcomes, such as the specific treatment protocols administered (e.g., surgery, chemotherapy with cycle counts, radiotherapy, and endocrine therapy) and key surgical results like breast‐conserving status (yes/no). Finally, the patient’s overall health and functional status were addressed by inventorying comorbidities (e.g., hypertension and diabetes mellitus) using a validated checklist [36] and objectively measuring functional capacity with the Barthel Index (0–100) to evaluate activities of daily living (such as feeding and mobility).

#### 3.3.2. Supportive Care Needs Evaluation

We selected the SCNS‐SF34 to assess supportive care needs. The SCNS‐SF34 has a 34‐item scale that evaluates five domains: physical and daily living needs, psychosocial needs, sexual health needs, health system and informational needs, and patient care and support needs [37]. Each item employs a 5‐point Likert scale (1 = “no need” to 5 = “high need”), with standardized domain scores (0–100) calculated using the formula:

(1) StandardizedScore=ΣRawScores−m×100m×k−1,

where *m* is the number of domain items and *k* = 5. Higher scores indicate greater unmet needs.

The SCNS‐SF34 was prioritized for three reasons. First, its multidimensional structure aligns with international consensus on core supportive care domains, enabling comprehensive evaluation of physiological, psychological, social, and informational needs. Second, the Chinese version has demonstrated strong reliability (Cronbach’s *α* > 0.75 across domains) and validity in breast cancer populations, ensuring cultural and clinical appropriateness [38]. Third, the standardized scoring system facilitates comparative analyses across heterogeneous patient subgroups and longitudinal studies. Compared to the full‐length SCNS (59 items), the SCNS‐SF34 reduces respondent burden—critical for elderly patients with fatigue, cognitive limitations, or reduced attention spans—while retaining comprehensive coverage of the same core dimensions as the full‐length version.

### 3.4. Data Process

#### 3.4.1. Features and Observations

Initial data preprocessing involved systematically excluding observations with null values to ensure analytical reliability and mitigate biases from incomplete records. This quality control procedure resulted in 157 of the 180 recruited participants meeting inclusion criteria.

Categorical variables were transformed into a set of binary dummy features, where each new feature effectively represents a ‘Yes/No’ status for a specific original variable. The original 27 variables expanded to 68 dimensions post‐transformation (Table 1). For example, the childbearing status variable was decomposed into four binary indicators: one child, two children, three or more children, and childless. This encoding enhanced algorithmic compatibility while preserving clinical interpretability, enabling precise identification of factors associated with elderly breast cancer patients’ supportive care needs.

**TABLE 1:** Top 10 features of observations (*n* = 157).

FtCode	FtName	FtType
Ft0	Age	Number
Ft1	Weight	Number
Ft2	Degree of fatigue	Ten Choises, From 0 to 10
Ft3	Whether breast‐conserving or not	Yes or No
Ft4	Whether metastasis has occurred or not	Yes or No
Ft5	Whether there are chronic diseases or not	Yes or No
Ft6	Whether there is heart disease or not	Yes or No
Ft7	Whether there is hypertension or not	Yes or No
Ft8	Whether there is diabetes or not	Yes or No
Ft9	Whether there is stroke or not	Yes or No

*Note:* (For the complete table, please refer to the Table A1).

Collectively, these preprocessing steps ensured dataset integrity and prepared it for subsequent machine learning applications.

#### 3.4.2. Statistical Characteristics

The final analytical cohort consisted of 157 female breast cancer patients (mean age: 66.6 ± 4.7 years), all with complete fundamental features and an SCNS‐SF34 score.

Anthropometric measurements showed moderate variability: average height = 160.0 ± 5.1 cm (range: 145–175 cm), weight = 64.7 ± 9.7 kg (range: 41–90 kg). Fatigue severity scores demonstrated significant dispersion (mean = 2.7 ± 2.4, median = 2, IQR:0–5), with some patients reporting maximum fatigue (9 on a 0–9 scale), indicating heterogeneous symptom burdens. The age distribution revealed right‐skewed clustering, with 75% of participants aged 70 years or younger. These baseline characteristics reflect a geriatric oncology population with typical age‐related comorbidities and treatment‐related symptom profiles (see Table 2).

**TABLE 2:** Overall statistical anthropometric descriptions of observations (*n* = 157).

Feature	Mean	Std	Min	25%	50%	75%	Max
Age	66.57	4.69	57	63	66	70	79
Height	159.97	5.14	145	157	160	163	175
Weight	64.66	9.65	41	57.5	64	70	90
Degree of fatigue	2.73	2.4	0	0	2	5	9

**FIGURE 2:**
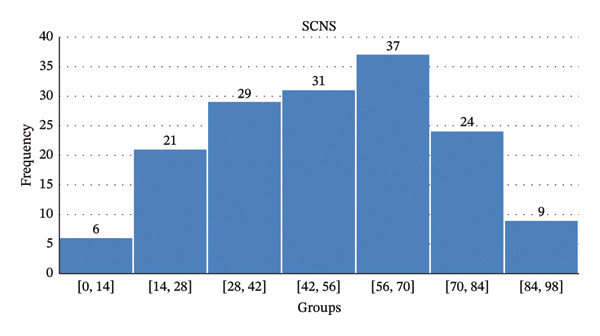
SCNS‐SF34 score distribution of observations (*n* = 157).

The histogram of SCNS‐SF34 scores (0–98 range) revealed a quasi‐normal distribution, peaking at the 56–70 interval (*n* = 37, 23.6% of 157 participants). Moderate needs (28–56) accounted for 38.2% (*n* = 60), while extreme needs (≥ 84) were infrequent (*n* = 9, 5.7%), indicating predominant mid‐to‐high unmet demands within this geriatric oncology cohort (see Figure 2).

The dummy variable analysis revealed heterogeneous distributions of supportive care needs across subgroups. Among 68 features, significant intergroup disparities (*p* < 0.05, Mann–Whitney U test) were observed in ethnicity, education, exercise frequency, and insurance type. Non‐Han ethnic groups exhibited 25.3% higher needs than Han counterparts (62.13 vs. 49.60), likely reflecting cultural barriers in care accessibility. Participants with primary‐level education reported elevated needs (53.18 ± 18.38 vs. 49.79 ± 22.29), suggesting health literacy gaps amplify demand for structured support. Paradoxically, low‐income individuals (0–1000 yuan) demonstrated lower needs (45.26 ± 22.76 vs. 52.23 ± 20.79), potentially indicating economic deprivation‐induced underreporting.

Notable clinical determinants included breast‐conserving surgery (53.61 ± 14.63) and sedentary behavior (56.17 ± 24.07), both associated with heightened needs. High variability in daily child visitation impact (SD = 23.02) implied qualitative differences in family support effectiveness. The findings underscore sociocultural and economic gradients as critical modulators of care needs, independent of treatment‐related factors (see Table 3).

**TABLE 3:** Top 10 SCNS distribution of dummy variables (*n* = 157).

FtCode	Yes‐[mean|std|count]	No‐[mean|std|count]
Ft3	53.61|14.63|11	50.38|21.87|136
Ft4	47.66|24.24|32	51.44|20.57|115
Ft5	48.8|21.98|81	52.85|20.59|66
Ft6	48.73|16.01|13	50.8|21.88|134
Ft7	48.43|21.1|67	52.46|21.59|80
Ft8	48.94|22.17|26	50.98|21.29|121
Ft9	38.39|nan|1	50.7|21.44|146
Ft11	62.13|21.2|12	49.6|21.18|135
Ft12	49.62|21.26|134	60.88|20.79|13
Ft13	49.48|21.9|41	51.06|21.28|106

*Note:* (For the complete table, please refer to the Table A2).

#### 3.4.3. Multicollinearity Assessment

Multicollinearity among features was assessed using the Variance Inflation Factor (VIF) [39], with adjustments to mitigate bias from dummy variables. When computing VIF for each feature, the dataset was dynamically filtered to retain the target feature while excluding other dummy variables from the same category—this avoids overestimating collinearity induced by related dummy variables [40]. VIF values were rounded to two decimal places and categorized into collinearity levels: No significant collinearity (VIF < 5), moderate collinearity (5 ≤ VIF < 10), or severe collinearity (VIF ≥ 10) [41] (see Table 4).

**TABLE 4:** Summary table of feature collinearity VIF analysis.

Collinearity level	VIF threshold	Number of features	Feature codes
No significant	VIF < 5	65	Ft0‐Ft12, Ft14‐Ft18, Ft21‐Ft67
Moderate	5 ≤ VIF < 10	3	Ft13, Ft19, Ft20
Severe	VIF ≥ 10	0	—

*Note:* (For the complete table, please refer to the Table A3).

VIF analysis was conducted to evaluate multicollinearity across 68 features. As shown in the summary table, the vast majority of features (65, 95.6%) exhibited no significant collinearity with VIF values below 5, indicating minimal linear correlation among most features. Only three features were categorized as having moderate collinearity (5 ≤ VIF < 10, range: 5.04–6.97), with no features exhibiting severe collinearity (VIF ≥ 10). Overall, the VIF results suggest that multicollinearity is not a critical issue in the dataset, supporting the suitability of all features for subsequent modeling [42].

### 3.5. Model Fit

In this research, survey‐derived data containing 68 variables were employed to predict SCNS‐SF34 scores. To validate the model performance, the dataset was randomly divided into a training subset and a validation subset. Given the small total sample size (*n* = 157), 10 cases (6.4% of the total sample) were allocated to the validation set to reserve more data for training—critical for capturing meaningful patterns in limited data. This approach reflects a trade‐off between ensuring the robustness of model training and maintaining the reliability of validation, which aligns with our core focus on interpretability rather than predictive efficacy. We acknowledge that the relatively limited sample size may constrain the generalizability of our findings, particularly for smaller subgroups. This limitation has been supplemented in the “Limitations” section.

Corresponding to this core focus, XGBoost parameters were selected to optimize model performance, with specific settings and rationales as follows:

• Objective: reg:squarederror—Suitable for regression tasks for predicting continuous SCNS‐SF34 total scores.
• Evaluation metric: Mean Absolute Error (MAE)—Robust to outliers, adapting well to the heterogeneous distribution of SCNS‐SF34 scores.
• Learning rate: 0.1—A moderate step size for gradient descent, balancing convergence speed and stability in small‐sample modeling;
• Max depth: 6—Limits decision tree complexity to reduce overfitting, a key concern in small‐dataset analysis.
• Subsample/colsample_bytree: 0.8—Randomly samples 80% of training data/features for each tree, reducing model variance and improving generalization.
• Regularization: reg_alpha = 0.1 (L1) and reg_lambda = 0.1 (L2)—Reduces overfitting risk and enhances model stability by controlling complexity.
• Boosting rounds: 150—Determined via trial and error to avoid underfitting or overfitting in the small‐sample context.

## 4. Results

### 4.1. Model Output

The XGBoost model demonstrated moderate predictive performance in estimating SCNS‐SF34 scores among elderly breast cancer patients, achieving an *R* ^2^ value of 0.58. This indicates the model explained 58% of the variance in these scores. Such performance suggests a reasonable fit, particularly considering the complex, multidimensional nature of the data and in stark contrast to a traditional linear regression model, which yielded an *R* ^2^ of only 0.017.

The XGBoost model yielded a MAE of 13.85, indicating the average absolute difference between predicted and actual SCNS‐SF34 scores. This level of error is considered acceptable, particularly in light of the patient population’s heterogeneity and the wide span of observed SCNS‐SF34 scores (ranging from 0 to 98, where an MAE of 13.85 constitutes approximately 14.1% of this total range). Furthermore, this MAE offers a practical benchmark for evaluating the model’s predictive utility in clinical settings. Deviations of this magnitude are potentially permissible for meaningful patient stratification into distinct care‐need categories (e.g., low, moderate, high), thereby aiding in resource allocation or tailored intervention planning.

### 4.2. Influencing Factors

#### 4.2.1. SHAP

SHAP analysis of the XGBoost model identified critical survey‐derived features (from 68) influencing supportive care needs. This enhanced model interpretability and pinpointed actionable targets for personalized interventions aimed at improving quality of life for elderly breast cancer patients.

#### 4.2.2. Factors and Influencing Mechanism

##### 4.2.2.1. Overall Analysis Based on SHAP

We identified the 10 most influential features driving supportive care needs in geriatric breast cancer patients. We calculated the mean absolute SHAP value for each feature as its importance:

(2) FeatureImportancei=Φi¯=1m∑j=1mΦixj,m∈OBS,

where *m* represents the total number of observations in the dataset, and *Φ*_*i*_(*x* ^(*j*)^) is the SHAP value for feature *i* of the *j*‐th observation.

The top‐ranked factors, sorted by SHAP feature importance, are in Table 5. These features collectively explain 78% of the variance in care needs, with fatigue severity and surgical treatment exhibiting the strongest positive associations, while higher weight and regular exercise demonstrated negative impacts (see Table 5).

**TABLE 5:** Top 10 important features based on SHAP (*n* = 157).

Feature name	FtCode	Feature importance
Degree of fatigue	Ft2	5.69
Weight	Ft1	3.49
Age	Ft0	1.99
Educational level_Junior high school	Ft15	1.57
Frequency of children’s visits_Daily	Ft38	1.57
Exercise situation_Three or four times a week	Ft56	1.31
Source of income_Pension	Ft53	1.3
Treatment plan_Surgery	Ft67	1.08
Economic income_0–1000 yuan	Ft45	1.07
Whether there is hypertension or not	Ft7	0.78

##### 4.2.2.2. Key Influencing Factors Analysis

**FIGURE 3:**
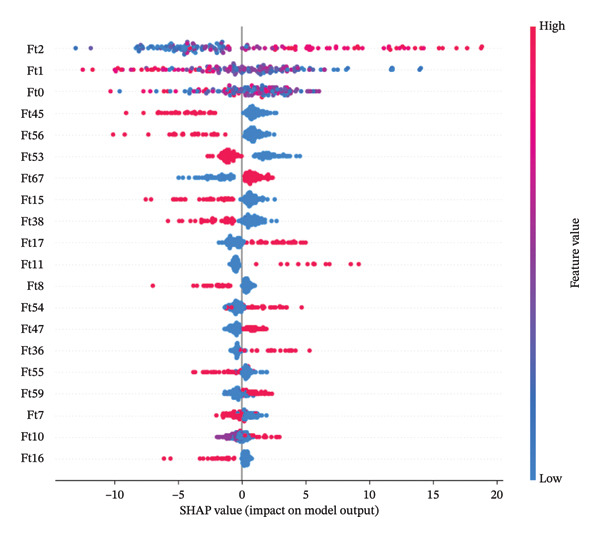
SHAP distribution summary of Top 20 Important Features (*n* = 157). (For the complete figure, please refer to the Figure A1).

The SHAP analysis revealed multidimensional determinants of supportive care needs in geriatric breast cancer patients, with demographic and physiological factors being predominant. Fatigue severity emerged as the strongest predictor of increased needs, likely due to its direct linkage to functional decline and the presence of comorbidities. Advanced age demonstrated nonlinear effects on predicted needs, potentially interacting with underlying frailty or individual resilience thresholds. A reduction in body weight correlated with heightened supportive care needs, possibly reflecting conditions such as malnutrition or sarcopenia, particularly in cachectic patients. Among treatment‐related factors, surgical intervention significantly amplified care demands, attributable to the challenges of postoperative recovery. Intriguingly, the presence of hypertension or diabetes was associated with lower supportive care needs. This counterintuitive finding may suggest that established disease management protocols and existing support systems for these chronic conditions could inadvertently mitigate or address a broader range of supportive care requirements, thus resulting in a lower overall reported need in this specific predictive context (see Figure 3).

Psychosocial factors exhibited nuanced influences on supportive care needs. Higher education (e.g., college attainment) and stable income sources like pensions were associated with mitigated demands, likely reflecting greater resource adequacy and self‐management capacity. The frequency of family support revealed a paradoxical effect: while daily visits from children appeared to reduce needs through direct assistance, sporadic visits correlated with exacerbated unmet demands. Similarly, economic deprivation (defined as income < 1000 yuan) paradoxically corresponded with lower expressed needs in the model, possibly due to underreporting or a resignation to limited care access. Exercise frequency further highlighted behavioral gradients: infrequent activity (1–2 times/month) was linked to increased needs, whereas regular exercise (≥ 1 time/week) appeared to improve self‐sufficiency and reduce predicted care demands.

These findings underscore the interplay of biological vulnerability, therapeutic burden, and socioeconomic inequity in shaping care demands. Targeted interventions should prioritize fatigue management, culturally sensitive care navigation, and structured support for low‐literacy populations.

Overall, the SHAP analysis revealed complex interactions between demographic‐physiological, treatment‐related, and psychosocial factors in determining supportive care needs.

## 5. Discussion

### 5.1. Baseline Results and Functional Patterns

This study identified multidimensional determinants of supportive care needs among elderly breast cancer patients through an interpretable machine learning framework. The XGBoost model demonstrated moderate predictive efficacy (*R* ^2^ = 0.58), outperforming traditional linear regression (*R* ^2^ = 0.017), which underscores the utility of nonlinear modeling in capturing complex interactions between clinical, demographic, and psychosocial variables [43]. Notably, fatigue severity emerged as the most influential predictor, aligning with prior evidence linking physical debilitation to heightened care demands in geriatric oncology populations [44]. These findings are consistent with systematic reviews indicating that supportive care needs in cancer patients are multidimensional, encompassing physical, psychological, informational, and social domains [8, 45]. Separately, our findings also highlighted paradoxical patterns, particularly concerning socioeconomic factors. For instance, economic deprivation (e.g., income < 1000 yuan) was associated with *lower* expressed needs in the model. This apparent paradox could stem from underreporting due to resignation to limited care access, or alternatively, reflect lower expectations of care quality among these low‐income groups.

### 5.2. Mechanistic Insights and Clinical Relevance

Through SHAP analysis, we gained critical insights into the complex drivers of supportive care needs among elderly breast cancer patients. This interpretable machine learning approach identified key predictive features and their impact patterns across physiological, treatment‐related, and psychosocial domains, revealing a multifaceted landscape of care determinants.

For the paradoxical finding that low‐income patients (0–1000 yuan) have lower expressed supportive care needs, this phenomenon could be explained by the priority ordering of needs driven by economic constraints. Low‐income families often prioritize limited economic resources to meet basic survival needs such as food, housing, and essential medical treatment; thus, the demand for supportive care (e.g., professional nursing, rehabilitation services, and psychological counseling) is relatively suppressed. This pattern of suppressed care needs among low‐income patients is also supported by existing research on socioeconomic disparities in health care demand reporting [46, 47]. An umbrella review on socioeconomic status and cancer confirmed that individuals with lower socioeconomic status face significant barriers in accessing comprehensive cancer care, including supportive services [48].

Physiological factors emerged as critical drivers. Fatigue severity and weight loss directly signaled functional decline, underscoring the need for targeted symptom management interventions [43]. Conversely, the reduced care needs associated with managed chronic conditions like hypertension and diabetes may reflect the efficacy of established care protocols, which potentially mitigate acute demands through standardized disease management. These findings align with evidence from CGA in older breast cancer patients, which emphasize the importance of integrated comorbidity management in reducing supportive care demands.

Treatment pathways and patient behaviors also presented significant influences. While surgical procedures often amplified immediate care demands due to postoperative recovery, patient‐driven actions such as regular exercise (≥ 1/week) were associated with significantly mitigated needs. This suggests that behavioral interventions encouraging physical activity could serve as valuable, potentially cost‐effective adjuncts to traditional clinical care. A systematic review and meta‐analysis confirmed that exercise interventions significantly reduce cancer‐related fatigue, with combined exercise programs showing the most robust effects [49].

Psychosocial gradients profoundly influenced care needs via sociocultural and economic factors. Low health literacy exacerbated unmet needs, likely by limiting access to care and health information. In contrast, pension‐based income and consistent family support buffered these demands, highlighting the protective effects of economic stability and social networks. This aligns with socioecological models of cancer care, emphasizing how individual capacity and structural inequities interact to shape care needs. Palliative and supportive care research confirms that social support and financial stability are critical determinants of care needs in older cancer patients [50].

Collectively, these patterns underscore the necessity of multidimensional approaches to address the diverse and interconnected factors influencing supportive care requirements in geriatric oncology populations. Such multidimensional interventions are widely endorsed for addressing the complex care needs of older cancer patients [51, 52].

### 5.3. Translational Intervention Priorities

Based on the key influencing factors identified in this study, we distill three clinically actionable intervention priorities to address unmet supportive care needs in elderly breast cancer patients: First, fatigue‐centric graded symptom management—screen patients using the 0–9 point fatigue scale combined with SCNS‐SF34 physical domain scores at admission or follow‐up; implement tiered care (mild fatigue: nurse‐led self‐management; moderate: multidisciplinary team intervention; severe: physician‐led drug treatment plus personalized rehabilitation); and conduct dynamic follow‐up (biweekly for 1–3 months postoperation, monthly for 3–6 months) to adjust plans. Second, optimize postoperative care processes—tailor interventions by phase: acute phase (hospitalization 1–7 days) focuses on pain control (target NRS score ≤ 3), early mobilization, and integrated chronic disease management; the recovery phase (1–3 months postdischarge) includes family caregiver training and structured exercise progression; and the maintenance phase (3–6 months postdischarge) leverages hospital‐community linkage to prevent fatigue recurrence. Third, enhanced psychosocial and clinical support—simplify health education for patients with low literacy and reinforce protective factors like consistent chronic disease management [53] (e.g., standardized hypertension medication adherence and regular nurse‐led monitoring) and regular family visits to buffer care demands. These priorities align with the interactive effects of physiological, treatment‐related, and psychosocial factors highlighted in our conclusions, laying a foundation for the subsequent Family‐Community‐Hospital collaborative care model.

### 5.4. Limitations and Methodological Considerations

Despite its contributions, this study has limitations. First, the sample size is relatively limited (*n* = 157), and all participants were recruited from a single specialized cancer hospital. This convenience sampling approach may limit the generalizability of the results, especially for smaller subgroups (e.g., non‐Han ethnic groups and rural patients) and patients from other regions of China. The small sample size also affects the statistical power of subgroup analyses, making it difficult to detect subtle differences in supportive care needs among specific subgroups.

Second, regarding the XGBoost model, although we have detailed the parameter settings and the rationale for selecting 10 cases as the validation set, the lack of cross‐validation may affect the reliability of the model’s predictive performance. The use of a single internal validation set (10 cases) may lead to overfitting to the specific sample, and the model’s performance may not be stably replicated in other populations. It should be emphasized again that the core objective of this study is to interpret the relationships between features and supportive care needs rather than to build a high‐precision predictive model, but the limitations of the model validation method still need to be acknowledged.

Third, the exclusive reliance on quantitative data may limit the understanding of psychosocial nuances. Quantitative data can only reflect the “surface” of supportive care needs (e.g., score levels) but cannot capture the subjective experiences and contextual factors behind the needs (e.g., the emotional distress of low‐income patients when they cannot afford supportive care services, and the cultural beliefs of ethnic minority patients that affect their perception of care needs). Qualitative data (e.g., in‐depth interviews and focus group discussions) would help to explore these nuanced factors and provide a more comprehensive understanding of supportive care needs.

Finally, the cross‐sectional design precludes causal inference; we can only observe the association between factors and supportive care needs at a specific time point but cannot determine the causal relationship (e.g., whether increased fatigue severity directly leads to higher supportive care needs or whether there are other mediating factors). Longitudinal studies are needed to trace temporal changes in care needs and clarify causal relationships.

### 5.5. Future Directions

Future research is crucial to advance the understanding and management of supportive care needs in elderly breast cancer patients, focusing on several key directions:

First, future studies will continue to recruit participants to increase the sample size and improve the representativeness of the sample. Additionally, larger samples will allow us to employ more robust validation techniques such as k‐fold cross‐validation to confirm the predictive stability and generalizability of the identified factor patterns, thereby benefiting the next phase of our study.

Second, longitudinal designs are essential to map the trajectories of care needs across different disease phases, capturing temporal changes and identifying critical periods for intervention.

Thirdly, intervention trials should test integrated care models that combine targeted strategies, such as personalized exercise programs for fatigue management, with health literacy interventions to empower patients in self‐care and decision‐making.

Finally, policy integration efforts must advocate for insurance reforms to expand coverage for supportive care services, particularly for economically vulnerable patients who face barriers to accessing essential care. By addressing these priorities, future research can bridge gaps in care delivery, promote health equity, and improve outcomes for elderly breast cancer patients.

## 6. Conclusion

This study, as a foundational component of our broader initiative to enhance the quality of life for elderly breast cancer patients in China, successfully identified key multidimensional determinants of their supportive care needs. By employing an interpretable machine learning framework, specifically XGBoost combined with SHAP analysis, we achieved moderate predictive performance (*R* ^2^ = 0.58) and, more importantly, gained nuanced insights into the complex interplay of factors influencing these needs—a significant advancement over traditional linear models.

Our findings revealed that physiological factors, particularly fatigue severity, are paramount drivers of increased supportive care demands. Treatment‐related aspects, such as surgical interventions, also substantially amplified needs. Intriguingly, psychosocial factors exhibited complex, sometimes paradoxical, influences: while resources like higher education, stable income, and consistent family support mitigated demands, economic deprivation appeared to suppress expressed needs, and sporadic family visits exacerbated them. Furthermore, managed chronic conditions like hypertension and diabetes were unexpectedly associated with lower predicted needs, potentially reflecting the impact of established care protocols.

The application of SHAP proved invaluable in dissecting these relationships, highlighting not only the most influential features but also their directional impacts and nonlinear patterns. This granular understanding of how demographic, physiological, treatment‐related, and psychosocial variables collectively shape supportive care requirements provides an empirical basis for developing more targeted and personalized care strategies.

While acknowledging the limitations inherent in our cross‐sectional design, convenience sample, and the use of a small internal validation set, this research lays crucial groundwork. The identified determinants and their mechanisms underscore the necessity for integrated care models. Future research, as outlined, should prioritize longitudinal studies to map need trajectories, intervention trials to test tailored supportive care strategies (e.g., for fatigue management and health literacy), and policy advocacy to improve access to care, especially for vulnerable populations.

Ultimately, by illuminating the multifaceted nature of supportive care needs, this study contributes vital knowledge towards our overarching goal of optimizing care outcomes and improving the well‐being of elderly breast cancer patients. The insights gained pave the way for subsequent phases of our research, including the development of assistive tools and collaborative care models designed to address these identified needs effectively.

## Funding

This work was supported by the Hospital‐Level Nursing Research Fund of Tianjin Medical University Cancer Institute and Hospital (Grant no. H2402).

## Conflicts of Interest

The authors declare no conflicts of interest.

## Figures and Tables

**Figure A1:**
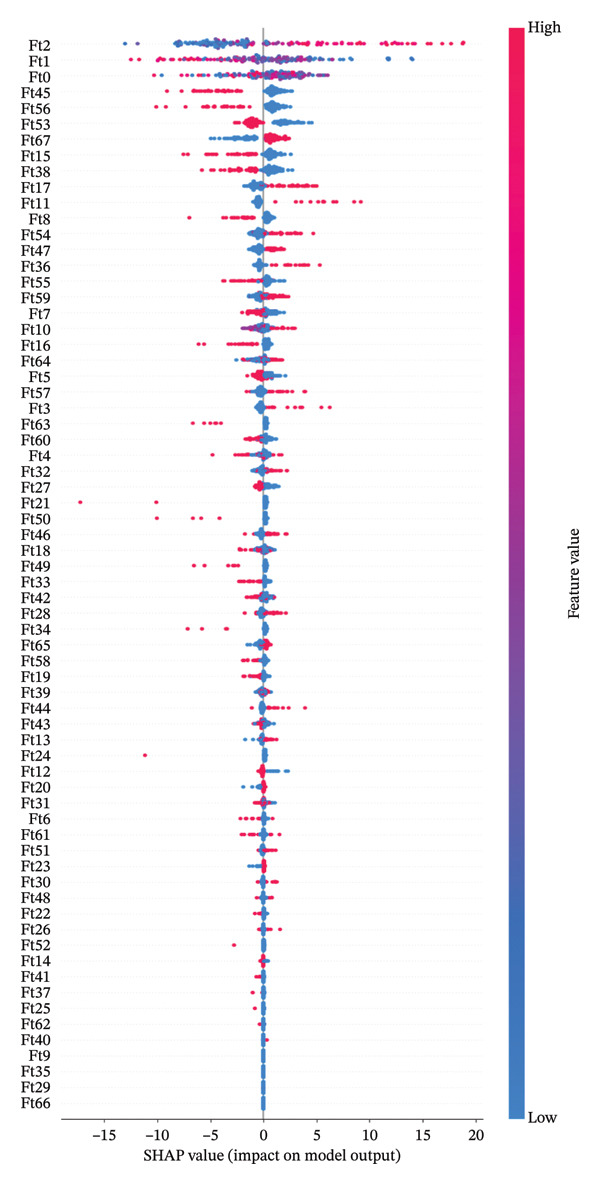
SHAP distribution summary of features (full graph).

## Data Availability

Research data are not shared.

## Appendix A

**Table A1:** Full features of observations (full table).

FtCode	FtName	FtType
Ft0	Age	Number
Ft1	Weight	Number
Ft2	Degree of fatigue	Ten Choises, From 0 to 10
Ft3	Whether breast‐conserving or not	Yes or No
Ft4	Whether metastasis has occurred or not	Yes or No
Ft5	Whether there are chronic diseases or not	Yes or No
Ft6	Whether there is heart disease or not	Yes or No
Ft7	Whether there is hypertension or not	Yes or No
Ft8	Whether there is diabetes or not	Yes or No
Ft9	Whether there is stroke or not	Yes or No
Ft10	Number of chronic diseases	Number
Ft11	Ethnic group_Others	Yes or No
Ft12	Ethnic group_Han	Yes or No
Ft13	Residence_Rural area	Yes or No
Ft14	Residence_Urban area	Yes or No
Ft15	Educational level_Junior high school	Yes or No
Ft16	Educational level_Junior college and above	Yes or No
Ft17	Educational level_Primary school and below	Yes or No
Ft18	Educational level_High school and technical secondary school	Yes or No
Ft19	Employment situation_Unemployed	Yes or No
Ft20	Employment situation_Retired	Yes or No
Ft21	Employment situation_Retired and re‐employed	Yes or No
Ft22	Marital status_Widowed	Yes or No
Ft23	Marital status_Married	Yes or No
Ft24	Marital status_Unmarried	Yes or No
Ft25	Marital status_Divorced or separated	Yes or No
Ft26	Living arrangement_Living with children	Yes or No
Ft27	Living arrangement_Living with spouse	Yes or No
Ft28	Living arrangement_Living with spouse and children	Yes or No
Ft29	Living arrangement_Others	Yes or No
Ft30	Living arrangement_Living alone	Yes or No
Ft31	Childbearing situation_One child	Yes or No
Ft32	Childbearing situation_Two children	Yes or No
Ft33	Childbearing situation_Three children or more	Yes or No
Ft34	Childbearing situation_Childless	Yes or No
Ft35	Frequency of children’s visits_Never	Yes or No
Ft36	Frequency of children’s visits_Occasionally	Yes or No
Ft37	Frequency of children’s visits_Rarely	Yes or No
Ft38	Frequency of children’s visits_Daily	Yes or No
Ft39	Frequency of children’s visits_Frequently	Yes or No
Ft40	Medical insurance payment_Public‐funded	Yes or No
Ft41	Medical insurance payment_Others	Yes or No
Ft42	Medical insurance payment_Urban residents	Yes or No
Ft43	Medical insurance payment_Urban employees	Yes or No
Ft44	Medical insurance payment_At one’s own expense	Yes or No
Ft45	Economic income_0–1000 yuan	Yes or No
Ft46	Economic income_1000–3000 yuan	Yes or No
Ft47	Economic income_3000–5000 yuan	Yes or No
Ft48	Economic income_More than 5000 yuan	Yes or No
Ft49	Source of income_Others	Yes or No
Ft50	Source of income_Re‐employment income	Yes or No
Ft51	Source of income_Supported by children	Yes or No
Ft52	Source of income_Government relief	Yes or No
Ft53	Source of income_Pension	Yes or No
Ft54	Exercise situation_Once or twice a month	Yes or No
Ft55	Exercise situation_Once or twice a week	Yes or No
Ft56	Exercise situation_Three or four times a week	Yes or No
Ft57	Exercise situation_Five times or more a week	Yes or No
Ft58	Exercise situation_Never exercise	Yes or No
Ft59	Disease stage_Stage I	Yes or No
Ft60	Disease stage_Stage II	Yes or No
Ft61	Disease stage_Stage III	Yes or No
Ft62	Disease stage_Stage IV	Yes or No
Ft63	Disease site_Bilateral	Yes or No
Ft64	Disease site_Right side	Yes or No
Ft65	Disease site_Left side	Yes or No
Ft66	Treatment plan_Chemotherapy (number of cycles)	Number
Ft67	Treatment plan_Surgery	Yes or No

**Table A2:** SCNS distribution of dummy variables (full table).

FtCode	Yes‐[mean|std|count]	No‐[mean|std|count]
Ft3	53.61|14.63|11	50.38|21.87|136
Ft4	47.66|24.24|32	51.44|20.57|115
Ft5	48.8|21.98|81	52.85|20.59|66
Ft6	48.73|16.01|13	50.8|21.88|134
Ft7	48.43|21.1|67	52.46|21.59|80
Ft8	48.94|22.17|26	50.98|21.29|121
Ft9	38.39|nan|1	50.7|21.44|146
Ft11	62.13|21.2|12	49.6|21.18|135
Ft12	49.62|21.26|134	60.88|20.79|13
Ft13	49.48|21.9|41	51.06|21.28|106
Ft14	50.82|21.23|105	50.13|22.03|42
Ft15	48.05|25.05|33	51.37|20.27|114
Ft16	52.04|20.67|24	50.34|21.6|123
Ft17	53.18|18.38|36	49.79|22.29|111
Ft18	49.98|21.7|53	50.98|21.32|94
Ft19	47.2|22.36|47	52.22|20.84|100
Ft20	52.38|20.61|97	47.2|22.64|50
Ft21	43.75|45.4|2	50.71|21.19|145
Ft22	51.75|15.98|17	50.47|22.04|130
Ft23	50.81|22.0|128	49.37|17.11|19
Ft24	17.57|nan|1	50.85|21.28|146
Ft25	40.63|nan|1	50.69|21.45|146
Ft26	51.32|14.93|13	50.55|21.96|134
Ft27	51.08|21.8|86	49.97|20.96|61
Ft28	48.41|24.06|33	51.26|20.62|114
Ft29	46.17|nan|1	50.65|21.46|146
Ft30	52.67|19.51|14	50.4|21.63|133
Ft31	50.42|20.34|78	50.85|22.66|69
Ft32	53.03|21.98|43	49.62|21.17|104
Ft33	48.88|22.8|22	50.93|21.21|125
Ft34	38.17|30.5|4	50.97|21.12|143
Ft35	38.4|nan|1	50.7|21.44|146
Ft36	58.61|21.4|18	49.5|21.23|129
Ft37	29.2|13.01|2	50.92|21.36|145
Ft38	47.85|23.02|44	51.8|20.66|103
Ft39	51.29|20.19|80	49.82|22.87|67
Ft40	89.87|nan|1	50.35|21.21|146
Ft41	48.74|16.72|5	50.69|21.58|142
Ft42	52.02|21.99|46	49.98|21.19|101
Ft43	49.25|20.06|77	52.13|22.81|70
Ft44	50.34|27.53|16	50.65|20.65|131
Ft45	45.26|22.76|34	52.23|20.79|113
Ft46	49.94|22.97|43	50.9|20.81|104
Ft47	53.43|20.83|53	49.03|21.64|94
Ft48	54.29|14.43|17	50.14|22.13|130
Ft49	34.96|22.9|6	51.29|21.15|141
Ft50	40.63|22.61|4	50.9|21.37|143
Ft51	51.03|20.2|31	50.51|21.78|116
Ft52	17.16|nan|1	50.85|21.28|146
Ft53	49.02|20.33|89	53.08|22.87|58
Ft54	56.17|24.07|35	48.88|20.28|112
Ft55	50.13|20.23|33	50.76|21.8|114
Ft56	43.44|18.68|25	52.09|21.68|122
Ft57	51.82|21.35|21	50.42|21.47|126
Ft58	49.9|20.97|33	50.83|21.6|114
Ft59	54.05|22.78|57	48.45|20.29|90
Ft60	48.18|20.15|53	52.0|22.04|94
Ft61	46.29|21.06|15	51.11|21.45|132
Ft62	50.12|15.22|5	50.64|21.61|142
Ft63	44.49|24.76|6	50.88|21.3|141
Ft64	48.93|21.88|65	51.96|21.03|82
Ft65	52.41|20.88|75	48.76|21.9|72
Ft66	74.8|nan|1	50.45|21.37|146
Ft67	52.4|20.81|96	47.27|22.25|51

**Table A3:** Variance inflation factor of features (full table).

FtCode	Variance inflation factor	Collinearity level
Ft0	1.7	No significant
Ft1	1.59	No significant
Ft2	1.57	No significant
Ft3	1.5	No significant
Ft4	1.9	No significant
Ft5	1.44	No significant
Ft6	1.43	No significant
Ft7	1.4	No significant
Ft8	1.76	No significant
Ft9	2.04	No significant
Ft10	1.5	No significant
Ft11	1.85	No significant
Ft12	1.87	No significant
Ft13	5.04	Moderate
Ft14	4.9	No significant
Ft15	1.79	No significant
Ft16	1.9	No significant
Ft17	2.97	No significant
Ft18	1.65	No significant
Ft19	6.97	Moderate
Ft20	5.94	Moderate
Ft21	1.3	No significant
Ft22	4.15	No significant
Ft23	4.94	No significant
Ft24	3.28	No significant
Ft25	1.94	No significant
Ft26	1.77	No significant
Ft27	2.16	No significant
Ft28	1.88	No significant
Ft29	2.14	No significant
Ft30	3.77	No significant
Ft31	2.55	No significant
Ft32	1.85	No significant
Ft33	1.8	No significant
Ft34	3.07	No significant
Ft35	2.16	No significant
Ft36	1.53	No significant
Ft37	2.56	No significant
Ft38	1.86	No significant
Ft39	1.64	No significant
Ft40	1.42	No significant
Ft41	1.58	No significant
Ft42	1.85	No significant
Ft43	2.77	No significant
Ft44	2.11	No significant
Ft45	3.4	No significant
Ft46	1.8	No significant
Ft47	2.09	No significant
Ft48	1.82	No significant
Ft49	1.74	No significant
Ft50	1.59	No significant
Ft51	3.39	No significant
Ft52	1.47	No significant
Ft53	4.76	No significant
Ft54	1.56	No significant
Ft55	1.4	No significant
Ft56	1.54	No significant
Ft57	1.62	No significant
Ft58	1.49	No significant
Ft59	1.86	No significant
Ft60	1.49	No significant
Ft61	1.79	No significant
Ft62	2.23	No significant
Ft63	2.39	No significant
Ft64	1.89	No significant
Ft65	1.72	No significant
Ft66	1.88	No significant
Ft67	2.12	No significant
